# A Point Prevalence Survey of Antibiotic Use in 18 Hospitals in Egypt

**DOI:** 10.3390/antibiotics3030450

**Published:** 2014-09-10

**Authors:** Maha Talaat, Tamer Saied, Amr Kandeel, Gehad A. Abo El-Ata, Amani El-Kholy, Soad Hafez, Ashraf Osman, Mohamed Abdel Razik, Ghada Ismail, Sherine El-Masry, Rami Galal, Mohamad Yehia, Amira Amer, David P. Calfee

**Affiliations:** 1 Global Disease Detection and Response Center, US Naval Medical Research Unit, No.3, Cairo 11517, Egypt; E-Mail: maha.talaat.ctr.eg@med.navy.mil; 2 Ministry of Health and Population, Cairo 11516, Egypt; E-Mails: kandeelamr@yahoo.com (A.K.); Ramygalal_81@yahoo.com (R.G.); 3 Cairo University Hospitals, Giza 11562, Egypt; E-Mails: gehadaboelata@yahoo.com (G.A.A.E.-A.); aaakholy@yahoo.com (A.E.-K.); 4 Alexandria University Hospitals, Alexandria 21111, Egypt; E-Mails: hafezsoad@gmail.com (S.H.); amira-amer@hotamil.com (A.A.); 5 Minya University Hospitals, Minya 61519, Egypt; E-Mails: ashrafosman31@yahoo.com (A.O.); mat_razek@hotmail.com (M.A.R.); 6 Ain Shams University Hospitals, Cairo 11591, Egypt; E-Mails: ghada.ismail@yahoo.com (G.I.); banou001@hotmail.com (S.E.-M.); 7 Azhar University Hospitals, Cairo 11517, Egypt; E-Mail: moh.yehya@hotmail.com; 8 Weil Cornell Medical College, New York, NY 4885, USA; E-Mail: dpc9003@med.cornell.edu

**Keywords:** antibiotic use, prevalence survey, surgical prophylaxis, Egypt

## Abstract

Inappropriate antibiotic use leads to increased risk of antibiotic resistance and other adverse outcomes. The objectives of the study were to determine the prevalence and characteristics of antibiotic use in Egyptian hospitals to identify opportunities for quality improvement. A point prevalence survey was conducted in 18 hospitals in March 2011. A total of 3408 patients were included and 59% received at least one antibiotic, with the most significant use among persons <12 years and intensive care unit patients (*p* < 0.05). Third generation cephalosporin were the most commonly prescribed antibiotics (28.7% of prescriptions). Reasons for antibiotic use included treatment of community—(27%) and healthcare-associated infections (11%) and surgical (39%) and medical (23%) prophylaxis. Among surgical prophylaxis recipients, only 28% of evaluable cases received the first dose within two hours before incision and only 25% of cases received surgical prophylaxis for <24 h. The prevalence of antibiotic use in Egyptian hospitals was high with obvious targets for antimicrobial stewardship activities including provision of antibiotic prescription guidelines and optimization of surgical and medical prophylaxis practices.

## 1. Introduction

Excessive and inappropriate use of antibiotics is highly associated with the emergence of antibiotic resistance [1], which presents a major threat to global public health. Antibiotic resistance reduces the effectiveness of and number of options for antibiotic treatment, leading to increased morbidity, mortality, and health care expenditures [2]. A recent prevalence survey in the United States found that 75% of hospitalized patients received more than one antimicrobial at the time of the survey [3] and studies have shown that a relatively large proportion of antibiotic use is inappropriate or unnecessary [4]. The European Surveillance of Antimicrobial Consumption Survey [5] found that the prevalence of antimicrobial use among patients in 172 hospitals in 25 European countries was 29% and identified several opportunities for improvement in antimicrobial prescribing practices [6]. Currently, there is little information available regarding hospital antibiotic use in the developing world. The objectives of this study were to describe the prevalence and characteristics of antibiotic use in Egyptian hospitals in order to provide benchmarking data and identify targets for quality improvement.

## 2. Experimental

### 2.1. Participating Hospitals

Hospitals included in the study were selected from among 37 Egyptian hospitals that were participating in a larger project to establish surveillance programs for healthcare-associated infections (HAIs) and antimicrobial resistance. For the antibiotic prevalence survey, a 50% sample was selected from among the 37 participating hospitals. Hospitals were stratified into two groups based on their participation status (*i.e.*, participant or non-participant) in an HAI surveillance pilot program. Hospitals were then randomly selected from each group for inclusion in the study.

### 2.2. Survey Methods

The methodology and definitions used for the prevalence survey were adopted from those used by the European Surveillance of Antimicrobial Consumption (ESAC) Project [5]. Data were collected using two standardized forms. One form collected data about each hospital (date of survey, patient population, ward type (medical, surgical, intensive care), and number of patients on each ward on the day of the survey. The second form was used to collect data about each patient included in the survey. The source of completing the patient data collection tool was through review of patient medical records and interviewing treating physicians. Information on patient demographics, antibiotic treatment including antibiotic name, dose, number of doses per day, and route of administration was obtained from the medical records.

Information on patient diagnosis group (body site for which antibiotics were being administered), and the indication of antibiotic use (treatment of community-acquired infection, treatment of hospital-acquired infection, medical prophylaxis, and surgical prophylaxis) were obtained from treating physicians. The medical records were examined for the presence of documentation of the indication of antibiotic use.

Additional information was collected from the records of patients receiving antibiotics for surgical prophylaxis. These data included timing of the first dose in relation to the timing of the surgical incision and the duration of prophylactic antibiotic administration as of the day of the survey.

Each participating hospital identified four persons with medical background to collect the survey data who were familiar with the hospital departments, wards and staff. They were either nurses, doctors or pharmacists. A specific day for data collection was pre-assigned for each hospital ward. All patients on a given hospital ward at 8:00 am on the day that the survey was scheduled were included in the survey. Data were collected on paper forms at each participating facility, reviewed for completeness by the director of the infection prevention and control team, and submitted to project coordinators at U.S. Naval Medical Research Unit No.3 (NAMRU3) in Cairo, where the data were entered into an electronic database for analysis. The study protocol was approved by the NAMRU3 Institutional Review Board as Non-research Protocol No. 1111—(DOD# namru3nr.2011.0011).

### 2.3. Statistical Analysis

Statistical analyses were conducted using STATA 11 (STATA Corporation, College Station, TX, USA). Differences in rates between groups were compared using the Chi-square test or Fisher’s exact test; *t*-test was used to compare continuous variables. Standard methods were used to calculate 95% confidence intervals (CIs) for the proportion of patients who received antibiotics in each hospital.

## 3. Results and Discussion

### 3.1. Results

**Figure 1:**
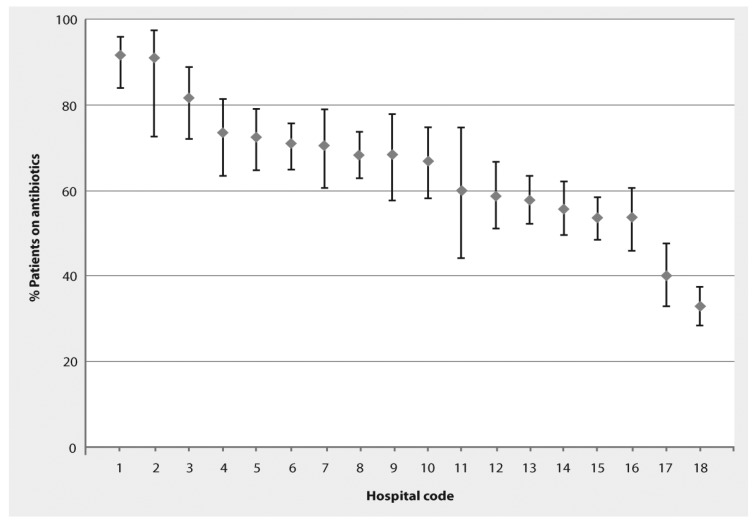
Percentage of patients in 18 Egyptian hospitals receiving antibiotics presented as actual measured percentage (diamond) with 95% confidence intervals (vertical lines).

**Figure 2:**
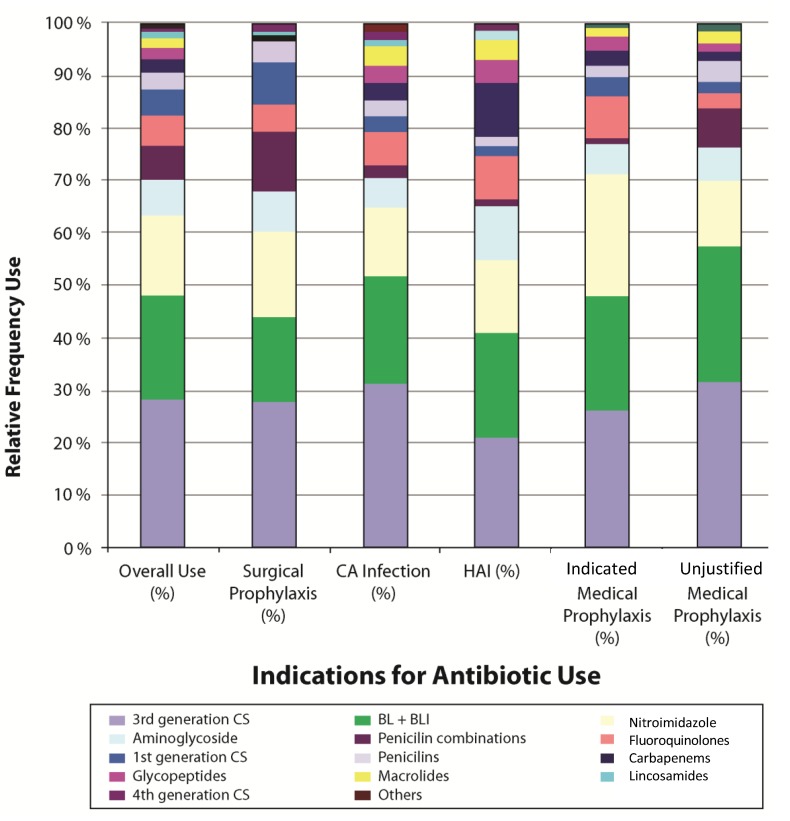
Frequency of use of individual antibiotic classes for prophylaxis and treatment among 3408 patients in 18 Egyptian hospitals—March 2011.

The study was completed during the period March 13–27, 2011. The 18 participating hospitals included five Ministry of Health and 13 university hospitals. Hospitals were classified as general hospitals (11), obstetrics and gynaecology (3), pediatric (2), general surgery (1) and orthopedic (1). The participating hospitals had a total of 7204 beds (mean 400 beds; median 292 beds; range 156–853 beds). Fifty-six intensive care units (39 adult, eight pediatric, and nine neonatal) were present in the 18 hospitals. Six of the hospitals (33%) reported that antibiotic use guidelines were available, but did not present the guidelines when asked for them. On the days of the survey, there were 3408 patients in the participating hospitals. Within the individual hospitals, the number of patients ranged from 23–447. A total of 2017 patients (59%) were receiving one or more antibiotics. The prevalence of antibiotic use in the participating hospitals ranged from 32.9%–91.7% (Figure 1). The 2017 treated patients received a total of 3194 antibiotic drugs (1.6 antibiotics per patient). The prevalence of combination therapy, defined as receipt of two or more antibiotics, was 28.4%. As shown in Figure 2, the most commonly prescribed antibiotics were broad-spectrum agents. Third generation cephalosporins were the most commonly prescribed antibiotics for all indications of antibiotic use, accounting for 28.7% of all antibiotic prescriptions. Penicillins with beta-lactamase inhibitors and mitronidazole derivatives accounted for 19.7% and 15.2%; respectively, and were almost equally prescribed for all indications (Figure 2).

The prevalence of antibiotic use was significantly more common among males, younger age groups, and intensive care unit patients (Table 1). The most common indication for antibiotic use, observed in 38.4% of antibiotic prescriptions, was surgical prophylaxis (Table 2). Other indications for antibiotic use included treatment of community-acquired infection (27.3%), medical prophylaxis (23%), and treatment of hospital-acquired infection (HAI) (11.3%). The relative frequency of each indication for antibiotic use varied among the different types of hospital locations. For example, the most common indication for antibiotic use among patients on medical wards was treatment of community-acquired infection (43.6%) whereas surgical prophylaxis was the most common indication for antibiotic use on surgical wards (66.5%). In intensive care units, 29.6% of antibiotics were administered for treatment of community-acquired infections and 27.3% were provided for hospital-acquired infections. Differences of antibiotic indications at various hospital locations were statistically significant. Among the 736 antibiotics given for medical prophylaxis, only 291 (39.5%) were given in association with a documented, medically-accepted indication for antibiotic prophylaxis.

Others include: Tetracyclins, Sulfonamides, Nitrofurans, antifungals, and second generation cephalosporins Bl + BLI: Drugs that have beta-lactam antibiotics (e.g., Penicillins or Cephalosporins) in addition to beta-lactamase inhibitor agents (sulbactam or clavulanic acid). Penicillin combinations: Drugs that have penicillin antibiotic combination to provide an extended spectrum of efficacy, particularly against penicillin-resistant infections (Ampicillin and cloxacillin combination).

**Table 1:** Prevalence of Antibiotic Use in 18 Egyptian Hospitals, March 2011.

Patient Characteristics		Total No. of Patients (*n* = 3408)	No. of Patients on Antibiotics	Prevalence of Antibiotic use (%)	*p*-Value
Sex	Male	1563	973	62.3	*p* < 0.001
	Female	1845	1044	56.6	
Age	<5 years	389	313	80.5	*p* < 0.01
	5–12 years	163	111	68.1	
	>12–65	2734	1521	55.6	
	>65	112	67	59.8	
	Surgical Ward	1572	979	62.3	
	Medical Ward	1409	713	50.6	

10 patient records had missing age.

**Table 2:** Indications for Antibiotic Use Stratified by Type of Hospital Ward.

Indications for Antibiotic Use	Antibiotic Prescriptions (number, (%))				*p*-value
	Medical wards *n* = 1127	Surgical wards *n* = 1470	Intensive care units *n* = 597	Total *n* = 3194	
Community-acquired infection	492 (43.6)	202 (13.7)	177 (29.6)	871 (27.3)	<0.01
Hospital-acquired infection	117 (10.4)	82 (5.6)	163 (27.3)	362 (11.3)	<0.01
Medical Prophylaxis					
Medically accepted indication for medical prophylaxis	200 (17.8)	23 (1.6)	68 (11.4)	291 (9.1)	<0.01
Unjustified indication for medical prophylaxis	149 (13.3)	186 (12.6)	110 (18.4)	445 (13.9)	<0.01
Surgical prophylaxis	169 (14.9)	977 (66.5)	79 (13.2)	1225 (38.4)	<0.01

The most common anatomical sites involved in the prophylactic use of antibiotics were the gynecologic tract (23.9%), gastrointestinal tract (16.3%), and skin, bone, and joints (14.8%) (Table 3). Among patients receiving antibiotics for treatment of infection, the most common anatomical sites of suspected or proven infection were the respiratory tract (39.2%), gastrointestinal tract (16%), and skin, bone and joints (15.7%) (Table 3).

**Table 3:** Distribution of Antibiotic Treatment and Prophylaxis by Diagnosis.

Diagnosis Group	Antibiotic Use		
	Total (*n* = 3194) No. (%)	Prophylaxis (*n* = 1961) No. (%)	Treatment (*n* = 1233) No. (%)
Respiratory tract	597 (18.7)	114 (5.8)	483 (39.2)
Gastrointestinal tract	516 (16.2)	319 (16.3)	197 (16.0)
Gynaecology	508 (15.9)	469 (23.9)	39 (3.2)
Skin, Bone & Joint	484 (15.2)	290 (14.8)	194 (15.7)
Central nervous system	232 (7.3)	188 (9.6)	44 (3.6)
No defined site	230 (7.2)	133 (6.7)	97 (7.8)
Cardiovascular system	217 (6.8)	174 (8.9)	43 (3.5)
Urinary tract	151 (4.7)	59 (3.0)	92 (7.5)
Immunology	147 (4.6)	127 (6.5)	20 (1.6)
Eye	63 (2.0)	55 (2.8)	8 (0.65)
Ear, nose, and throat	49 (1.5)	33 (1.7)	16 (1.3)

Out of 1572 patients in the surgery wards, 802 patients (51%) received antibiotics for surgical prophylaxis. Among these 802 patients, 702 (87.5%) had information in their medical charts regarding the start time of the prophylactic antibiotic(s). The duration of antibiotic prophylaxis, however, could be determined for only 333 patients (41.5%) (Table 4). Out of 702 patients who received surgical prophylaxis, 156 (22.2%) received an antibiotic more than 2 h before the surgical incision, 252 (35.9%) received the antibiotic within two hours prior to the incision, and 294 (41.9%) received the antibiotic after the surgical incision had been made. The percentage of surgical antibiotic prophylaxis courses initiated more than 2 h before the incision varied greatly among the different surgical specialties, ranging from 0%–55.6%, and was highest in the burn and plastic surgery departments. Out of 333 surgeries with a known duration for surgical prophylaxis, 18 (5.4%) were prescribed a single dose of antibiotics, whereas 73.6% of patients were prescribed antibiotics for more than 24 h.

**Table 4:** Timing of First Dose and Duration of Surgical Antibiotic Prophylaxis.

Type of surgery	No. of operations with known start time	>2 h before incision No. (%)	≤2 h before incision No. (%)	After incision No. (%)	No. of operations with known duration of antibiotic prophylaxis	Single dose No. (%)	≤24 h after incision No. (%)	>24 h after incision No. (%)
Cardiothoracic surgery	43	10 (23.3)	14 (32.5)	19 (44.2)	29	0	0	29 (100)
ENT surgery	28	5 (17.9)	16 (57.1)	7 (25)	11	0	2 (18.2)	9 (81.8)
Burns and plastic surgery	9	5 (55.6)	1 (11.1)	3 (33.3)	2	0	0	2 (100)
General surgery	122	37 (30.3)	35 (28.7)	50 (41.0)	67	1 (1.5)	12 (17.9)	54 (80.6)
Neurosurgery	41	12 (29.3)	2 (4.9)	27 (65.9)	4	0	0	4 (100)
OB/GYN	267	44 (16.5)	99 (37.1)	124 (46.4)	76	11 (14.5)	49 (64.5)	16 (21.1)
Ophthalmology	40	2 (5.0)	16 (40.0)	22 (55.0)	40	2(5.0)	2(5.0)	36 (90.0)
Orthopedic surgery	83	22 (26.5)	46 (55.4)	15 (18.1)	74	4 (5.3)	5(6.8)	65 (87.9)
Urology	22	4 (18.2)	8 (36.4)	10 (45.5)	11	0	0	11 (100)
Vascular surgery	5	0	5 (100)	0	6	0	0	6 (100)
Other *	42	15 (35.7)	10 (23.8)	17 (40.5)	13	0	0	13 (100)
Total	702	156 (22.2)	252 (35.9)	294 (41.9)	333	18 (5.4)	70 (21.0)	245 (73.6)

* Other include: Face and jaw surgeries, thyroid surgeries, and male genital surgeries.

### 3.2. Discussion

To our knowledge, this is the first large scale assessment of antibiotic use practices outside of North America and Europe. In this project, we applied the same survey method for the assessment of the prevalence of and indications for antibiotic use as that used by the European Surveillance of Antimicrobial Consumption (ESAC) Project [5] to characterize the use of antibiotic agents in hospitals in Egypt. The ESAC methodology including the time frame and the census of the patients on the day of the survey was applicable and feasible and the data collection tools were simple and easy to use. While the European survey was completed using a web-based format, this study used a paper-based format in order to allow participation by facilities in which access to the internet was limited, or not available. This and other minor modifications may allow this methodology to be used in other countries and regions, including resource-limited areas, in which the prevalence of antibiotic use has not yet been characterized.

This study identified that 59% of patients in the participating Egyptian hospitals were receiving one or more antibiotic agents at the time of survey completion. This is substantially higher than the prevalence of antibiotic use reported in similar studies performed in Europe and the US [6,7,8]. Although the prevalence of antibiotic use was quite variable among participating hospitals, ranging from 32.9%–91.7%, all of the participating hospitals exceeded the 29% prevalence reported in the 2009 ESAC Survey conducted in 172 hospitals representing 29 European countries [6]. It is important to note that the differences in the prevalence of antibiotic use do not necessarily indicate that there is more inappropriate use of antibiotic agents in Egyptian hospitals. Some of these differences may be due to differences in patient populations or in the prevalence of infectious diseases among hospitalized patients or to the inclusion of a larger proportion of teaching hospitals among our participating hospitals. Our data do suggest, however, that not all of the differences can be attributed to such population differences. In fact, although specific data regarding appropriateness of individual antibiotic prescriptions were not collected, the survey data have identified a number of opportunities for improvement in antibiotic use practices. International antibiotic use guidelines were known to clinicians in only a limited number of hospitals. The translation of such guidelines into active antibiotic use policies could lead to improvements in antibiotic use practices, which could result in reductions in overall antibiotic use and its associated complications within these hospitals. 

Evaluation of the specific indications for antibiotic use revealed several specific opportunities for improvement. Surgical prophylaxis was the most common indication for antibiotic administration reported, accounting for 38.4% of all antibiotic prescriptions. A review of the details of surgical prophylaxis prescribing revealed that the selection, timing, and duration of administration were frequently inconsistent with the evidence-based practices recommended in Europe and the US [9]. For example, the first dose of antibiotic was administered either more than 2 h before incision or after incision in 64% of patients receiving antibiotics for surgical prophylaxis. In addition, 74% of patients received prolonged courses of prophylactic antibiotic therapy (*i.e.*, more than 24 h). Recent European studies have also demonstrated that prolonged administration of antibiotics for surgical prophylaxis is common. In the 2009 ESAC Survey, surgical antibiotic prophylaxis was administered for >24 h in 53% of patients [6] whereas this type of prolonged prophylaxis was observed in 21% patients receiving surgical prophylaxis in 38 French hospitals [8]. Regarding choice of therapy, first-generation cephalosporins, penicillins, and vancomycin accounted for only 12% of all antibiotic agents given for surgical prophylaxis even though these are the recommended agents for a large proportion of surgical procedures. Broad-spectrum agents such as third-generation cephalosporins and beta-lactam plus beta-lactamase inhibitor combinations, on the other hand, accounted for 44% of surgical prophylaxis prescriptions, which might have serious implications on the emergence of multidrug-resistant organisms. Informal discussions with clinicians provided some misconceptions, e.g., prolonging the duration of antibiotic prophylaxis and selecting a broad-spectrum agent for prophylaxis were practices commonly used to reduce the risk of surgical site infections and other healthcare-associated infections in the post-operative period. In response to these findings, a quality improvement project focused on improving surgical site infection prevention practices, including surgical antibiotic prophylaxis, is currently being implemented in several Egyptian hospitals.

We also identified that a large proportion of patients receiving antibiotics for “medical prophylaxis” had no justification of a medically-accepted indication for prophylaxis. This group of patients accounted for 14% of all antibiotic use and 60% of patients who were receiving antibiotic therapy for the purpose of medical prophylaxis. Discussions with clinicians suggested that it was relatively common practice in some of the participating hospitals for patients to be given antibiotics during their hospital stay to reduce the risk of acquiring healthcare-associated infection. This suggests that efforts to strengthen basic infection prevention practices would eventually improve clinicians’ confidence in infection prevention and control program which could result in substantial reductions in the use of antibiotic agents and the associated complications among hospitalized patients. 

Although this study provides novel data that will be useful for quality improvement initiatives in individual hospitals and for the Egyptian healthcare system in general, a few limitations should be noted. First, data were collected from only 5% of the hospitals in Egypt. The data may therefore not be representative of antibiotic use in all hospitals throughout the country. In addition, the data were collected from a convenience sample rather than a random sample of Egyptian hospitals. The distribution of hospital types among those that participated in the survey is quite different from the overall distribution of hospital types in Egypt. We sampled approximately 30% of the university hospitals but less than 2.5% of the Ministry of Health hospitals. Finally, although all persons involved in data collection had completed a formal training program prior to participating in the survey, there was no central validation of the submitted data.

## 4. Conclusions

In conclusion, this is the first study to quantify and characterize antibiotic use practices in hospitals in Egypt. The information gained from this point prevalence survey has helped to prioritize limited resources by allowing identification of several specific opportunities to improve antibiotic use practices that may result in improved patient outcomes and lower healthcare costs within Egyptian hospitals. Although the data and their implications have largely been presented and discussed here in aggregate form up to the national level, data from the individual participating hospitals may prove to be even more useful for identifying more specific opportunities for quality improvement within these hospitals. We intend to repeat the survey in order to assess changes in the prevalence and characteristics of antibiotic use in Egyptian hospitals over time, as we thought that the point prevalence survey implemented was an efficient and simple method for assessing antibiotic use in Egypt.

## Disclaimer

The views expressed in this article are those of the author and do not necessarily reflect the official policy or position of the Department of the Navy, Department of Defense, the U.S. Government nor the Egyptian Ministry of Health and Population. This work was supported by US Agency for International Development in Egypt (USAID/Egypt), Work Unit 263-P-00-10-00005-00. The study protocol #1111 was approved by the U.S. Naval Medical Research Unit No. 3 Institutional Review Board in compliance with all applicable Federal regulations governing the protection of human subjects. Authors Maha Talaat and Tamer Saied are contractors of the U.S. Government. This work was prepared as part of their official duties. Title 17 U.S.C. §105 provides that ‘Copyright protection under this title is not available for any work of the United States Government.’ Title 17 U.S.C. §101 defines a U.S. Government work as a work prepared by a military service member or employee of the U.S. Government as part of that person’s official duties.
